# Supplementary material for: HIVprotI: an integrated web based platform for prediction and design of HIV proteins inhibitors
Source: J Cheminform. 2018 Mar 9;10:12. doi: 10.1186/s13321-018-0266-y (PMC5845081; doi:10.1186/s13321-018-0266-y)
Supplement: Supplementary file 2 — Additional file 2. Source code of HIVProtI web server. [file 13321_2018_266_MOESM2_ESM.zip › HIVprotI_Source-code/hivprotI/weka-3-6-6/doc/weka/clusterers/forOPTICSAndDBScan/Utils/UpdateQueueElement.html]

UpdateQueueElement


---


|  |  |  |
| --- | --- | --- |
| **Overview** \| **Package** \| **Class** \| **Tree** \| **Deprecated** \| **Index** \| **Help** \| **Weka's home** | |  |
| **PREV CLASS**   NEXT CLASS | **FRAMES**    **NO FRAMES**     **All Classes** |  |
| SUMMARY: NESTED \| FIELD \| CONSTR \| METHOD | DETAIL: FIELD \| CONSTR \| METHOD |  |


---


## weka.clusterers.forOPTICSAndDBScan.Utils Class UpdateQueueElement

```
java.lang.Object
  weka.clusterers.forOPTICSAndDBScan.Utils.UpdateQueueElement
```

**All Implemented Interfaces:**: RevisionHandler

---

``` public class UpdateQueueElement extends java.lang.Object implements RevisionHandler ```

UpdateQueueElement.java

**Version:**
:   $Revision: 1.3 $

**Author:**
:   Matthias Schubert (schubert@dbs.ifi.lmu.de), Zhanna Melnikova-Albrecht (melnikov@cip.ifi.lmu.de), Rainer Holzmann (holzmann@cip.ifi.lmu.de)

---

**Constructor Summary**

`UpdateQueueElement(double priority,
java.lang.Object o,
java.lang.String objectKey)`


| **Method Summary** | |
| --- | --- |
| `java.lang.Object` | `getObject()`             Returns the object |
| `java.lang.String` | `getObjectKey()`             Returns the key |
| `double` | `getPriority()`             Returns the priority for this object |
| `java.lang.String` | `getRevision()`             Returns the revision string. |

**Methods inherited from class java.lang.Object**

`equals, getClass, hashCode, notify, notifyAll, toString, wait, wait, wait`

**Constructor Detail**

### UpdateQueueElement

```
public UpdateQueueElement(double priority,
                          java.lang.Object o,
                          java.lang.String objectKey)
```


**Method Detail**

### getPriority

```
public double getPriority()
```

:   Returns the priority for this object

    :   **Returns:**: priority

---


### getObject

```
public java.lang.Object getObject()
```

:   Returns the object

    :   **Returns:**

---


### getObjectKey

```
public java.lang.String getObjectKey()
```

:   Returns the key

    :   **Returns:**: objectKey

---


### getRevision

```
public java.lang.String getRevision()
```

:   Returns the revision string.

    :   **Specified by:**: `getRevision` in interface `RevisionHandler`
    :   **Returns:**: the revision


---


|  |  |  |
| --- | --- | --- |
| **Overview** \| **Package** \| **Class** \| **Tree** \| **Deprecated** \| **Index** \| **Help** \| **Weka's home** | |  |
| **PREV CLASS**   NEXT CLASS | **FRAMES**    **NO FRAMES**     **All Classes** |  |
| SUMMARY: NESTED \| FIELD \| CONSTR \| METHOD | DETAIL: FIELD \| CONSTR \| METHOD |  |


---
